# Development of a Worldwide Consortium on Evolutionary Participatory Breeding in Quinoa

**DOI:** 10.3389/fpls.2016.00608

**Published:** 2016-05-09

**Authors:** Kevin M. Murphy, Didier Bazile, Julianne Kellogg, Maryam Rahmanian

**Affiliations:** ^1^Sustainable Seed Systems Lab, Department of Crop and Soil Sciences, Washington State University, PullmanWA, USA; ^2^Unité Propre de Recherche Gestion des Ressources Renouvelables et Environnement, Department of Environment and Societies, French Agricultural Research and International Cooperation OrganizationMontpellier, France; ^3^Centre for Sustainable DevelopmentTehran, Iran

**Keywords:** evolutionary participatory breeding, *Chenopodium quinoa*, global network, population variety, agrobiodiversity

## Abstract

*Chenopodium quinoa* is gaining global importance due to its excellent protein quality and tolerance of abiotic stresses. The last 60 years have seen major strides in the expansion of quinoa crop production and experimentation. Quinoa’s wide genetic diversity has led to its agronomic versatility and adaptation to different soil types, particularly saline soils, and environments with extremely variable conditions in terms of humidity, altitude, and temperature. The potential of quinoa to contribute to global food security was recognized in 2013 in the declaration of the International Year of Quinoa (IYQ). Promoting the use of improved homogeneous quinoa varieties standardized to comply with applicable norms on seeds or suited to intensified conventional agriculture farming systems may not generate the necessary resilience needed to respond to current and future global challenges. Maintaining and increasing quinoa biodiversity is imperative, as the dynamics of the global expansion of quinoa may constitute a threat to farmers if the spread is generated with a narrow genetic base. In this article, we propose that the method of evolutionary participatory breeding could be a useful tool to develop new quinoa genetic material in cooperation with farmers. We introduce preliminary results on quinoa population development with farmers in the Pacific Northwest region of the USA. We conclude that a global collaborative network on quinoa (GCN-Quinoa) could be the baseline for participatory plant breeding programs originating in developing or developed countries to meet the needs of farmers across a diversity of agronomic systems and a wide range of physical environments.

## Quinoa as a Keystone Protein Crop for Global Food Security

*Chenopodium quinoa* Willd., a cultivated species of the Amaranthaceae family grown for its edible and highly nutritious achene, represents a neglected and underutilized species (NUS; Kahane et al., 2013). Quinoa is a gynomonoecious allotetraploid (2*n* = 4× = 36) and a facultative autogamous annual with outcrossing from 0.5 to 17.36% (Gandarillas, 1979; Silvestri and Gil, 2000). Protein content of quinoa typically ranges from 12 to 17%, and is influenced by factors such as cultivar, soil fertility and environment (Rojas et al., 2015). Most notably, quinoa has a well-balanced complement of all essential amino acids, giving it a protein quality superior to that of other crop species (Repo-Carrasco et al., 2003). Quinoa also lacks gluten and therefore can be safely eaten by people with gluten allergies or celiac disease (Zevallos et al., 2015).

Andean farmers of South America took the first steps in domesticating quinoa from its wild forms more than 5,000 years ago (Planella et al., 2015). Seed exchanges and migrations of human populations played an important role in the early development of landraces with indehiscent, larger seeds, adaptations to drought and saline soils, and varietal differences in photoperiod, altitude, and diverse rainfall conditions (Bazile et al., 2013). Five ecotypes are globally recognized: Inter-Andean valleys (Colombia, Ecuador, and Peru); Altiplano (northern highlands in Peru and Bolivia); Yunga (Bolivia); Salares (salt flats or southern highlands in Bolivia, Chile and Argentina); and Coastal (coastal or sea level areas in central and southern Chile; Risi and Galwey, 1984; Fuentes et al., 2012; Bazile et al., 2013). Long-term selection in these sub-centers of quinoa biodiversity confers specific and often unique traits of adaptation to the landraces. Diversity is observed in a wide array of colors of plants and seeds, and through differences in branching patterns and panicle types, grain productivity, abiotic stress tolerances and disease resistance (Fuentes and Bhargava, 2011; Ruiz-Carrasco et al., 2011). Even if the potential adaptation to climate change inherent in quinoa confers probable utilization for agricultural development in other parts of the world, a better understanding of the diversity of the actual contexts of cultivation in its area of origin is crucial in order to predict where specific quinoa ecotypes would have the most agronomic impact (Jacobsen et al., 2015). Quinoa has higher drought and salinity tolerance than many other crops, and tremendous variation in these traits are evident among quinoa ecotypes and existing quinoa varieties and landraces (Bazile et al., 2015; Peterson and Murphy, 2015a). For example, the Salares ecotype is best suited to withstand extreme conditions of this cold high-altitude desert at 4.000 m.a.s.l. This ecotype is extremely drought resistant and adapted to saline and sandy soils surrounding the salt lakes. Alternatively, the quinoa of the Coastal ecotype grows from sea level up to 1500 m.a.s.l. and possesses characteristics specific to this unique environment; specifically, adaptation to high annual precipitation, resistant to pre-harvest sprouting, high evapotranspiration index, a comparatively high degree of heat tolerance, and salinity tolerance.

Due to quinoa’s increasing popularity and recent expansion of cultivated area on a global scale, farmers and plant scientists are emphasizing morphological and physiological modifications that allow for mechanized harvesting, improved heat tolerance and downy mildew resistance, and reduced agricultural inputs (Bazile et al., 2015; Bonifacio et al., 2015). New sources of heat tolerance or disease resistance genes from wild relatives are being introgressed into commercial varieties by plant breeders; however, undesirable characteristics such as seed dormancy and shattering from the donor species can decelerate a quinoa-breeding program (Zurita-Silva et al., 2014). The objective of this long-term participatory development project is to implement and successfully conduct a collaborative European-North American EPB program for quinoa with field sites in the host countries and in South America as well as across several countries in Africa and Asia.

## Participatory Plant Breeding

Participatory variety selection (PVS) and Participatory plant breeding (PPB) have been practiced in various regions and on various crops over the last 30 or more years. Much of the focus has been in developing countries, but there has also been some work in developed countries, often with a focus on low-input and/or organic agriculture (Chable et al., 2008; Ghaouti et al., 2008; Leroy et al., 2014; Campanelli et al., 2015). The primary difference between PPB and PVS is the extent of farmer participation in the various stages of the breeding program. PVS involves testing and selecting new varieties developed by the institutional system within farmers’ fields and at local research stations in various environments and allowing farmers to compare these varieties with local farmer varieties. In a PPB program, farmers are routinely involved in decision-making throughout the entire breeding process and not just in the final testing of advanced breeding lines. Trials are conducted in farmers’ fields and managed by farmers. Farmers score varieties according to criteria that they define. Data generated by breeders provides the basis for the selection of successful varieties by farmers, in dialog with breeders.

On marginal lands, and in relation to NUS and non-commercial crops, formal plant breeding has made less of an impact (Ceccarelli et al., 2009). To address this, plant breeders working on a diversity of crops in various regions began involving farmers in setting breeding objectives and in the subsequent selection and testing of breeding materials. This direct dialog benefits the breeding process with possible farmer participation in setting objectives, creating variability, selecting and testing breeding material, and producing and diffusing seed of new cultivars (Weltzien and Christinck, 2008). The objective of PPB research was to more appropriately respond to the specific needs of under-represented farmers and to optimize production, adoption, and food sovereignty through improved local adaptation of cultivars to relevant abiotic and biotic pressures, as well as to prevalent cultural norms (Ceccarelli et al., 2001; Sperling et al., 2001; Weltzien et al., 2003). Improvement of PPB continues as researchers identify different factors that affect breeding material performance including number of entries to be evaluated (Thiele et al., 1997; Ceccarelli et al., 2000), gender (Kamara et al., 1996; Sthapit et al., 1996; Weltzien et al., 1996), independent versus paired or group evaluations, and inclusion of on-station evaluations by farmers (McElhinny et al., 2007).

Among the important outcomes of PPB is the positive impact it has on biodiversity. PPB is a highly decentralized process where research occurs in a multitude of farmers’ fields in different ecoregions and microclimates. PPB produces resilient varieties with high levels of phenotypic plasticity that often differ across locations depending on local climate and ecosystems, as well as social, cultural and economic factors influencing farmer preferences. With regard to quinoa, this diversity can be easily observed *in situ* in farmers’ fields showing a wide array of colors in plants and seeds, different types of branching and panicles, as well as having variation in grain productivity, abiotic stress tolerance and disease resistance (Bazile et al., 2013). Varieties selected by farmers are often genetically variable, similar to traditional farmer landraces, in stark contrast to the majority of varieties produced by conventional breeding in which all the plants are genetically identical. The fact that farmers’ varieties are not genetically uniform is precisely what makes them resilient to a variety of stresses that are made more unpredictable by climate change.

## Evolutionary Participatory Breeding (EPB)

Due to the predominance of monocultures in global crop production, crop diversity occurs at a scale where individual genotype unit areas (GUAs; Mundt and Browning, 1985) are often many square kilometers (Newton et al., 2009). The genetic uniformity inherent in monocultures can restrict the crop’s ability to tolerate diverse abiotic environmental stresses, pests and diseases, thereby leading to a potential decrease in the stability of the cropping system (Hooper et al., 2005; Hughes et al., 2008). Chakraborty and Newton (2011) suggest that strategies for establishing greater resilience and improved yield stability should focus on the introduction of increased genetic variability, both within and between cultivars, into agricultural systems. Such crop populations are better equipped to adapt to future unpredictable temporal climate shifts than are monocultures (Louafi et al., 2013). Genetically uniform cultivars have been shown to lack the ability to adjust and adapt to highly unpredictable environmental fluctuations, in direction and range, and novel stress factors (Verboom et al., 2010). Evolutionary breeding provides a framework with which to address current and predicted threats to agriculture as a result of climate change and agricultural intensification.

Evolutionary breeding involves four distinct stages: (i) creation of genetic diversity; (ii) multiplication of seeds from each cross followed by an equal mixing of populations if more than one cross will be used in a given population; (iii) repeated sowing and harvesting of the population in one or more agronomic environment without active selection of individual plants; and (iv) use of the seed in culturally relevant settings as food or feed, or use of the seed as a basis for further breeding (Döring et al., 2011). EPB merges the evolutionary breeding method described by Suneson (1956), Allard and Adams (1969), Allard (1988) and Phillips and Wolfe (2005) with farmer participatory breeding to develop high-yielding, disease-resistant cultivars of desired quality while maintaining a high degree of genetic variation to allow for adaptability to fluctuations in environmental conditions (Murphy et al., 2005; Döring et al., 2011; Murphy et al., 2013; Ruiz et al., 2014; Bazile, 2015).

## Quinoa Populations and Preliminary EPB Methodology

Ten biparental quinoa populations were developed at Washington State University using seed from the USDA National Genetic Resources Program and from private seed companies in the US (**Table 1**) (Peterson et al., 2015). Parents were chosen based on one or more of the following characteristics from results of several years of preliminary testing: seed yield, day-neutral photoperiod, resistance to downy mildew, heat tolerance, drought tolerance, seed size, plant height, tolerance to lodging, mold and pre-harvest sprouting resistance, and plant and seed color. Seed was advanced to the F_3_ and populations were planted on two organic farms, located in Pullman and Quilcene, in 2014, each representing a distinct location and associated agronomic challenges (Washington State, USA). Pullman represents a very hot and dry climate in the major wheat growing region of the Pacific Northwest. Quilcene represents a cool, coastal climate with where late season rains caused significant yield loss due to pre-harvest sprouting and downy mildew (*Peronospora variabilis*) in 2010 and 2013.

**Table 1:** Pedigree and seed yield of 10 populations in two locations (Pullman: High-heat environment and Quilcene: Low-heat environment) in Washington State, USA in 2014.

Population designation	Female parent	Male parent	Seed Yield (g/400 m^2^)	
			High-heat environment	Low-heat environment
QUP11WA-101	Biobio	Colorado 407D	7.5	12579
QUP11WA-102	Colorado 407D	QQ74	94.9	15215
QUP11WA-103	Cherry Vanilla	Black	1.3	10994
QUP11WA-104	Kaslaea	QQ74	8.5	9371
QUP11WA-105	QQ065	QQ74	7.8	5132
QUP11WA-106	QQ065	Black	20.8	6215
QUP11WA-107	QQ74	Black	10.1	3864
QUP11WA-108	QQ74	Cherry Vanilla	no seed	5009
QUP11WA-109	Temuko	Biobio	no seed	4113
QUP11WA-110	Oro de Valle	Black	7.3	4305

No human selection was conducted on the F_3_ quinoa populations in 2014, the hottest year on record in eastern Washington. Extremely low population yields in Pullman reflect the effect of heat on pollen viability (**Table 1**). Variety trials, which included the majority the parents of the populations, adjacent to the population trials yielded no seed at all in any of the varieties. Population 102, derived from two relatively heat tolerant parents (Colorado 407D and QQ74) showed the highest seed yield (**Table 1**). The populations in Quilcene were not subject to heat stress, nor stress due to excessive rainfall, and subsequently seed yield was much higher. This evolutionary breeding method was repeated with F_4_ seed in 2015 in Quilcene, however, instead of a bulk harvest, 200 panicles from each population were harvested individually after a deliberate and prolonged exposure to rainy early autumn conditions to encourage pre-harvest sprouting in the populations. Additionally, farmers from a range of farms in the Olympic Peninsula conducted both negative and positive selection on six populations on a nearby organic farm in Chimacum, Washington. Some individual seed from each positively panicle will be saved and replanted in pedigree headrows in 2016, but the majority of the F_5_ seed will be bulked and the populations reconstituted; this seed will be distributed to collaborators in partner countries to begin the EPB process in 2016.

## Perspectives on a GCN-Quinoa

**FIGURE 1:**
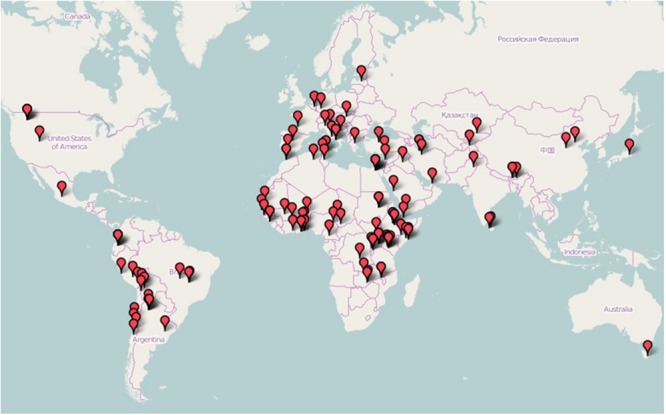
**Global distribution of the current members of the pilot Global Collaborative Network on Quinoa (GCN-Quinoa)**.

The pilot GCN-Quinoa is the first step in moving forward with EPB in quinoa. After just one year of existence, more than 150 researchers are part of this global network and represent a diversity of people from 60 countries who currently share a similar curiosity in adapting quinoa to their region (**Figure 1**). Nowadays, the number of research centers with experimental quinoa programs is increasing worldwide, even in regions that are not yet producing or consuming quinoa (Bazile et al., 2015; Peterson and Murphy, 2015b). However, the primary quinoa research centers are located in countries where quinoa has been grown traditionally for centuries. As a result, there exists a significant gap in varietal, agronomic, and cultural knowledge of quinoa production and consumption in Africa for example. In that context, the main objective of the GCN-Quinoa is to create a collaborative space to facilitate exchanges between producers, processors, distributors, politicians, and all persons involved in quinoa development for the promotion and sustainable use of quinoa genetic resources. Access and benefit sharing about quinoa seeds are of importance that is why a specific forum of discussion was generated online on this topic. Members of the GCN-quinoa interested in testing quinoa through EPB approach have joined, and can continue to join, the development of the Worldwide Consortium on Evolutionary Participatory Breeding in Quinoa and benefit from the expertise and support of the group.

Increasing consumer demand in quinoa and the usefulness of the crop in adapting to climate change means that the cultivation of quinoa is undergoing rapid expansion worldwide. It thus acknowledges the role that quinoa’s biodiversity and high nutritional value could play in providing global food security. Since this minor crop could become a major crop, how can we continue the varietal improvement efforts that farmers in the Andean countries have been carrying out for generations? The achievement of this goal requires innovative approaches to plant breeding in order to avoid the well-known risks of genetic uniformity and vulnerability as well as to encourage sustainable agriculture with a decreased use of agrochemical inputs.

How does one breed for farmer’s needs? Involving a large number of farmers in evaluation and selection throughout the selection process is an excellent way to take into account heterogeneity across environments (Kotschi, 2010). This form of decentralized selection is critical in involving and empowering farmers from the beginning of the breeding process. There is a need for a paradigm shift in agriculture which must enhance the role of agrobiodiversity and the need for innovation in plant breeding is particularly crucial (Ceccarelli, 2009). Increasing genetic diversity in quinoa populations may be a relevant method to adapt crops to environmental changes, in particular, to agricultural areas where environmental conditions are marginal for crop production. It will be important to consider market options, and the desire among end-users for a uniform product, when developing populations. Therefore, particular attention must be given to the selection toward uniformity of targeted quality traits, such as seed size and color, which can be identified visually post-harvest, and protein content and starch characteristics, which can be evaluated in a lab (Wu et al., 2014).

Evolutionary PPB with quinoa has the potential to contribute significantly to sustainable agriculture if a lasting connection can be made among all the relevant stakeholders. Linking researchers at the global level who are involved with quinoa trials in a collaborative network may be the most effective pathway. One of the challenges offered during the (IYQ, 2013) was to devise a means and methodology to increase the likelihood of quinoa’s success in the introduction and development in non-traditional quinoa growing regions. Next-generation quinoa research will require a collaborative effort from a dedicated group of experts who possess the ability to evaluate the diverse global needs of quinoa research and to subsequently conduct this research in a cooperative manner (Bazile, 2013).

Given that quinoa cultivation and research is expanding to parts of world that are not familiar with the crop, researchers and farmers would benefit from being included in a network with other researchers facing similar challenges. They would also benefit from access to a wide range of germplasm which is currently concentrated in gene banks in Latin America, as noted above, and to which researchers in other parts of the world may not have ready access (Bazile et al., 2016). The International Treaty on Plant Genetic Resources for Food and Agriculture (the Treaty) proposes the implementation of a multilateral system (MLS) of exchange of genetic resources for both facilitate access to genetic resources and ensure benefit-sharing. The Treaty provides a monitoring of genetic resources with the harmonized material transfer agreements; certification of origin to prevent biopiracy and the disclosure of origin to share the benefits. With few notable exceptions (Argentina, Bolivia, and Colombia), Andean countries^1^ have ratified the Treaty. Under its MLS, countries agree to make available their genetic resources stored in collections that are under their control and in the public domain for 64 crops that are listed in Annex 1 of the Treaty. Although some discussions are under way to possibly enlarge this list, quinoa does not currently belong to this Annex 1 which means that, so far, the rules of the MLS do not apply for the exchanges of quinoa genetic material. The Convention on Biological Diversity (CBD) does only regulate bilateral access and benefit sharing, but this is difficult to apply to quinoa as the crop is now planted internationally, not restricted to the Andean region, and this has been the case for decades. For quinoa, there is no single existing legal framework providing a comprehensive coverage of all the issues related to the genetic resources and their sustainable management (Chevarria-Lazo et al., 2015). Considering that the status of quinoa is now rapidly changing from a NUS to a major crop for food and agriculture, the opportunity exists to question current dominant agricultural models and plant breeding methods and begin to move forward toward more participatory approaches in order to address the real needs of the end-users in a context of global changes (Anvar, 2008; Trommetter, 2010). Research and plant breeding in quinoa should be based on more flexible genetic material with the potential to maintain yield stability while continually evolving in response to changes in climate. Considering the GCN-Quinoa as a vector to disseminate evolutionary material, considered as open seed sources, with a high level of genetic diversity will certainly favor adaptation in new environments. Nevertheless, without direct dialog resulting from close collaborations between farmers and researchers, we may struggle to reach the next step for quinoa introduction to cropping systems for local diets.

## Author Contributions

KM wrote two sections of, and edited the entire manuscript. KM initiated the quinoa populations and along with JK set up and conducted the participatory breeding research. JK provided edits to the entire manuscript. DB and MR contributed significantly to the writing and editing of the manuscript. In addition, DB. initiated the Global Collaborative Network on Quinoa.

## Conflict of Interest Statement

The authors declare that the research was conducted in the absence of any commercial or financial relationships that could be construed as a potential conflict of interest.

The reviewer RP and handling Editor declared their shared affiliation, and the handling Editor states that the process nevertheless met the standards of a fair and objective review.

## Footnotes

1 Contracting Parties: Chile (2016), Ecuador (2004), Peru (2003), Venezuela (2005); Only Signatorie: Argentina (2002), Colombia (2002); Non Contracting parties: Bolivia.
